# High *HSPB1* expression predicts poor clinical outcomes and correlates with breast cancer metastasis

**DOI:** 10.1186/s12885-023-10983-3

**Published:** 2023-06-03

**Authors:** Qin Huo, Juan Wang, Ni Xie

**Affiliations:** 1 Biobank, Shenzhen Institute of Translational Medicine, Shenzhen Second People’s Hospital, First Affiliated Hospital of Shenzhen University, Health Science Center, Shenzhen University, 518035 Shenzhen, China; 2 Department of General Practice, Army Medical Center of PLA, Chongqing, 400042 China

**Keywords:** *HSPB1*, Breast cancer, Prognostic biomarker, Cell proliferation, Metastasis

## Abstract

**Background:**

Heat shock protein beta-1 (*HSPB1*) is a crucial biomarker for pathological processes in various cancers. However, the clinical value and function of *HSPB1* in breast cancer has not been extensively explored. Therefore, we adopted a systematic and comprehensive approach to investigate the correlation between *HSPB1* expression and clinicopathological features of breast cancer, as well as determine its prognostic value. We also examined the effects of *HSPB1* on cell proliferation, invasion, apoptosis, and metastasis.

**Methods:**

We investigated the expression of *HSPB1* in patients with breast cancer using The Cancer Genome Atlas and immunohistochemistry. Chi-squared test and Wilcoxon signed-rank test were used to examine the relationship between *HSPB1* expression and clinicopathological characteristics.

**Results:**

We observed that *HSPB1* expression was significantly correlated with the stage N, pathologic stages, as well as estrogen and progesterone receptors. Furthermore, high *HSPB1* expression resulted in a poor prognosis for overall survival, relapse-free survival, and distant metastasis-free survival. Multivariable analysis showed that patients with poor survival outcomes had higher tumor, node, metastasis, and pathologic stages. Pathway analysis of *HSPB1* and the altered neighboring genes suggested that *HSPB1* is involved in the epithelial-to-mesenchymal transition. Functional analysis revealed showed that transient knockdown of *HSPB1* inhibited the cell migration/invasion ability and promoted apoptosis.

**Conclusions:**

*HSPB1* may be involved in breast cancer metastasis. Collectively, our study demonstrated that *HSPB1* has prognostic value for clinical outcomes and may serve as a therapeutic biomarker for breast cancer.

**Supplementary Information:**

The online version contains supplementary material available at 10.1186/s12885-023-10983-3.

## Background

Breast cancer is one of the most common malignant tumors in women. The incidence rate is rising each year, which has a significant negative impact on women's health and quality of life [1–3]. Despite advancements in current treatment techniques, including surgery and chemotherapy, the outcome of breast cancer remains unsatisfactory [4]. Therefore, finding novel molecular indicators for breast cancer are urgently required.

Heat shock protein 27, also referred to as heat shock protein beta-1 (HSPB1), belongs to the small HSP family. Its purpose is to stop or prevent cellular proteins from denaturing or unfolding in response to stress or elevated temperatures [5]. *HSPB1* regulates many pathological processes in cancer, including drug resistance, apoptosis, and metastasis [6–8]. *HSPB1* is considered an important molecular target for tumor growth inhibition and apoptosis induction [9] and is vital in the regulation of tumorigenesis and the development of some cancers [10–12]. For example, esophageal squamous cell carcinoma with overexpression of *HSPB1* has a worse prognosis [13]. Upregulation of *HSPB1* is related to poor overall survival in hepatocellular carcinoma and promotes tumorigenesis [14]. Further research has revealed that *HSPB1* knockout led to a decrease in insulin levels and expression of growth factor-like binding protein 2, which may promote the proliferation and metastasis of hepatocellular carcinoma [15]. Moreover, *HSPB1* expression is related to the epithelial-mesenchymal transition (EMT). Through the upregulation of *Snail1* and *PRRX1*, *HSPB1* overexpression promotes EMT and drives the migration and invasion of salivary adenoid cystic carcinoma cells [16]. Additionally, *HSPB1* interferes with bone metastasis in breast cancer [17] and modulates the *PTEN* levels in human breast cancer cells [18]. The potential relevance of *HSPB1* and its underlying mechanisms in the development of breast cancer have yet to be elucidated.

While the relationship between *HSPB1* and tumorigenesis has been demonstrated, limited evidence has illustrated the clinical significance and function of *HSPB1* in breast cancer. In this study, we used a systematic and comprehensive approach to assess the relationship between *HSPB1* expression and clinicopathological characteristics in patients with breast cancer. We also determined the prognostic value of *HSPB1*. Additionally, we examined the effects of *HSPB1* on cell proliferation, invasion, apoptosis, and metastasis. We believe that this study has identified *HSPB1* as a therapeutic target for breast cancer.

## Methods

### Patient tissue specimens

A total of 18 cancerous tissues (*n* = 15) and normal adjacent tissue (*n* = 3) were collected from patients undergoing surgery for breast cancer at the First Affiliated Hospital of Shenzhen University from April 2019 to February 2021. These samples were taken from tissues removed surgically. In addition, we collected 20 samples of in situ breast cancer and lung metastases. The tissues were quickly stored at − 80 °C until use, according to the Tumor Bank protocol [19]. Written informed consent was provided by all participants.

### *HSPB1* expression and univariate/multivariate regression analysis

We obtained data with the relevant clinical characteristics from The Cancer Genome Atlas (TCGA). TCGA is an open-access resource, that includes 33 types of cancer from approximately 20,000 patients [20]. These data were used for *HSPB1* expression analysis as well as preliminary analyses of univariate logistic regression and multivariate Cox regression to examine prognostic factors and clinical outcomes, including overall survival (OS), disease-specific survival (DSS), and progress free interval (PFI). Patients with breast cancer were examined to determine whether *HSPB1* expression was correlated with clinicopathological variables to better understand the prognostic value of *HSPB1* based on status (tumor or normal), patient age (≤ 60 or > 60 years of age), tumor (T) stage (T1, T2, T3, or T4), node (N) stage (N0, N1, N2, or N3), metastasis (M) stage (M0 or M1), pathological stage (stage I, stage II, or stage III), estrogen receptors (ER; negative or positive), progesterone receptors (PR; negative or positive), and human epidermal growth factor receptor 2 (HER2; negative or positive). We examined the association between *HSPB1* expression and clinical characteristics using Chi-square and Wilcoxon signed-rank tests.

### Cell culture, cell transfection and quantitative real-time PCR

We obtained six cell lines from the Chinese Academy of Sciences, including human normal mammary epithelial cells: MCF10A, and all breast cancer cell line types: luminal A: T47D, luminal B: BT474, HER-2 positive: SK-BR-3, Triple-negative A: MDA-MB-453, Triple-negative B: MDA-MB-231. A mix of 10% fetal bovine serum (FBS) and RPMI Medium 1640 (DMEM; Gibco, Carlsbad, CA, USA) was used to culture each cell line. Two sequences targeting *HSPB1* siRNAs were synthesized by Gene Pharma (Suzhou, China): si-1: 5′-GCCAUUAUUAGAGACCUCATT-3′ and si-2: 5′-UCACCAUCCCAGUCACCUUTT-3′. Cells were transfected using Lipofectamine 2000 (Invitrogen, United States). The Pure Link RNA Mini Kit (Invitrogen, United States) was used to extract total RNA. Quantitative real-time PCR (RT-PCR) was performed under the following conditions: 95 °C for 5 min, and 40 cycles of 95 °C for 30 s, 60 °C for 45 s and 72 °C for 30 s. Primers were purchased from Gene Pharma (Suzhou, China) using the following sequences: *HSPB1*, 5′-CTCTGAAGGGTCCGAAGTGAT-3′ and 5′-ATTCCTGTGGTGGTCCAAAAC-3′; Actin: 5′-CACCATTGGCAATGAGCGGTTC-3′ and 5′-AGGTCTTTGCGGATGTCCACGT-3′.

### Western blot and immunohistochemical analysis

Samples were tested for total protein concentration using the bicinchoninic acid method. A 12% SDS-PAGE gel was used to separate 20 µg of each protein sample. We then transferred the separated proteins onto polyvinylidene fluoride membranes for primary antibody detection. The membranes were then incubated for 1 h at room temperature with alkaline phosphatase-conjugated secondary antibodies (Roche, Switzerland), followed by three washes with Tris-buffered saline with Tween 20 for 15 min. Primary antibodies against *HSPB1* (1: 1000 dilution), glyceraldehyde-3-phosphate dehydrogenase (1: 5,000 dilution), vimentin (1: 1000 dilution), N-cadherin (1: 1000 dilution) and E-cadherin (1: 1000 dilution) were used. All the antibodies used in this study were purchased from CST (USA). The labeled proteins were visualized via chemiluminescent imaging.

Immunohistochemical assays were performed on human breast cancer and adjacent tissues. All fresh tissues were cryopreserved before being processed into histology blocks for sectioning. Briefly, after deparaffinization and rehydration, antigen retrieval was performed by heating 5μm -thick sections at 95 °C for 15 min in 10 mM citrate buffer (pH 6.0). After incubation with the primary antibody against *HSPB1* (1: 1000 dilution) for 12 h, the sections were counterstained with hematoxylin to label the nuclei. Two pathologists blindly evaluated and scored all stained sections to determine the degree of immunostaining.

### Kaplan–Meier plotter and PrognoScan database analysis

We analyzed the correlation between *HSPB1* transcription levels and OS, relapse-free survival (RFS), and distant metastasis-free survival (DMFS) in patients with breast cancer using the Kaplan–Meier plotter database (http://www.kmplot.com/) [21]. For all tests, the hazard ratios (HR) with 95% confidence intervals were defined as significant at *p* < 0.05. The sample sizes of the high- and low-HSPB1 groups were 939 and 940 for OS, 2,465 and 2,464 for RFS, and 1,383 and 1,382 for DMFS, respectively.

The PrognoScan database (http://dna00.bio.kyutech.ac.jp/PrognoScan/index.html) [22] was used to analyze the correlation between *HSPB1* expression and prognosis in patients with breast cancer, including OS, RFS, and DMFS (these data from the GSE1456-GPL96 and GSE2990 cohorts). Samples were divided into groups of high or low *HSPB1* expression levels, with the lowest 50% and the top 50% considered low and high expression, respectively.

### Analysis of *HSPB1-*interacting genes and proteins

The gene–gene interaction network and protein–protein interaction network of *HSPB1* were constructed using GeneMANIA (http://www.genemania.org) and STRING online (https://string-db.org/) [23, 24]. To verify the correlation between *HSPB1* and the altered neighboring genes, a breast cancer cohort from the TCGA database was analyzed using bc-GenExMinerv 4.8 (http://bcgenex.ico.unicancer.fr/BC-GEM/GEM-Accueil.php?js=1) [25] and RNA-seq data (N = 4,712). Using the GSCALite database (http://bioinfo.life.hust.edu.cn/web/GSCALite/) [26], pathways were analyzed for the altered neighboring genes.

### Cell viability and colony formation assay

We tested the viability of cells using a Cell Counting Kit-8 (CCK8; Dojindo, Kumamoto, Japan) in accordance with the manufacturer's protocol. In 96-well plates, culture media (100μL) and 3,000 cells were plated. After culturing for 0 to 72 h at 37 °C in a humidified incubator with 5% CO_2_, each well was incubated for 2 h with 10 μL of CCK-8 reagent. Measurement of absorbance at 450 nm and assessment of proliferation ability were conducted using a spectrophotometer (Bio-Rad Laboratories, CA, USA). The colony-forming potential of all cancer cells was assessed by seeding them onto six-well plates at a density of 300 cells/well. After 10–14 days of culture, cells were removed from the medium, fixed with 4% paraformaldehyde, stained with 0.1% crystal violet, and washed thrice with phosphate buffered saline (PBS) before imaging.

### Wound healing and Transwell® invasion assays

Transfected SK-BR-3 and MDA-MB-231 cells (si-NC and si*HSPB1*) were seeded and cultured in an FBS containing medium at 37 °C until 100% confluence. A straight scratch was made through each culture using a 200 μL pipette tip. After wounding, cells were washed three times with PBS and then replenished with fresh serum-free media. The wound area was photographed immediately (t = 0 h) and after 72 h (t = 72 h) using an inverted microscope.

Transwell® chambers coated with Matrigel were filled with a 200 μL cell suspension in serum-free medium. The lower chamber was filled with 600 μL of complete medium. Subsequently, the plates were incubated for 24 h. By optical microscopy, we analyzed the migrated cells by fixing them with 4% paraformaldehyde and staining them with 0.1% crystal violet.

### Analyses of cell apoptosis

A culture plate was seeded with cells and grown to 70% confluence. For the cell apoptosis assay, SK-BR-3 and MDA-MB-231 cells were stained with 5 µL Annexin V-fluorescein isothiocyanate and 5 µL propidium iodide. A FACS-can flow cytometer and Cell Quest software were used for the follow-up analysis (Becton Dickinson, USA).

### Statistical analysis

All statistical analyses were conducted using R version 3.6.3. The Wilcoxon rank sum test was used to analyze the difference in *HSPB1* expression between normal (*n* = 113) and tumor tissues (*n* = 1,119). We examined the relationship between *HSPB1* expression and clinicopathological characteristics using the Chi-squared test and Wilcoxon signed-rank test. We used Kaplan–Meier to evaluate the prognostic value of *HSPB1* expression. All tests were defined as significant at *p* < 0.05.

## Results

### HSPB1 expression increased in patients with breast cancer

**Fig. 1:**
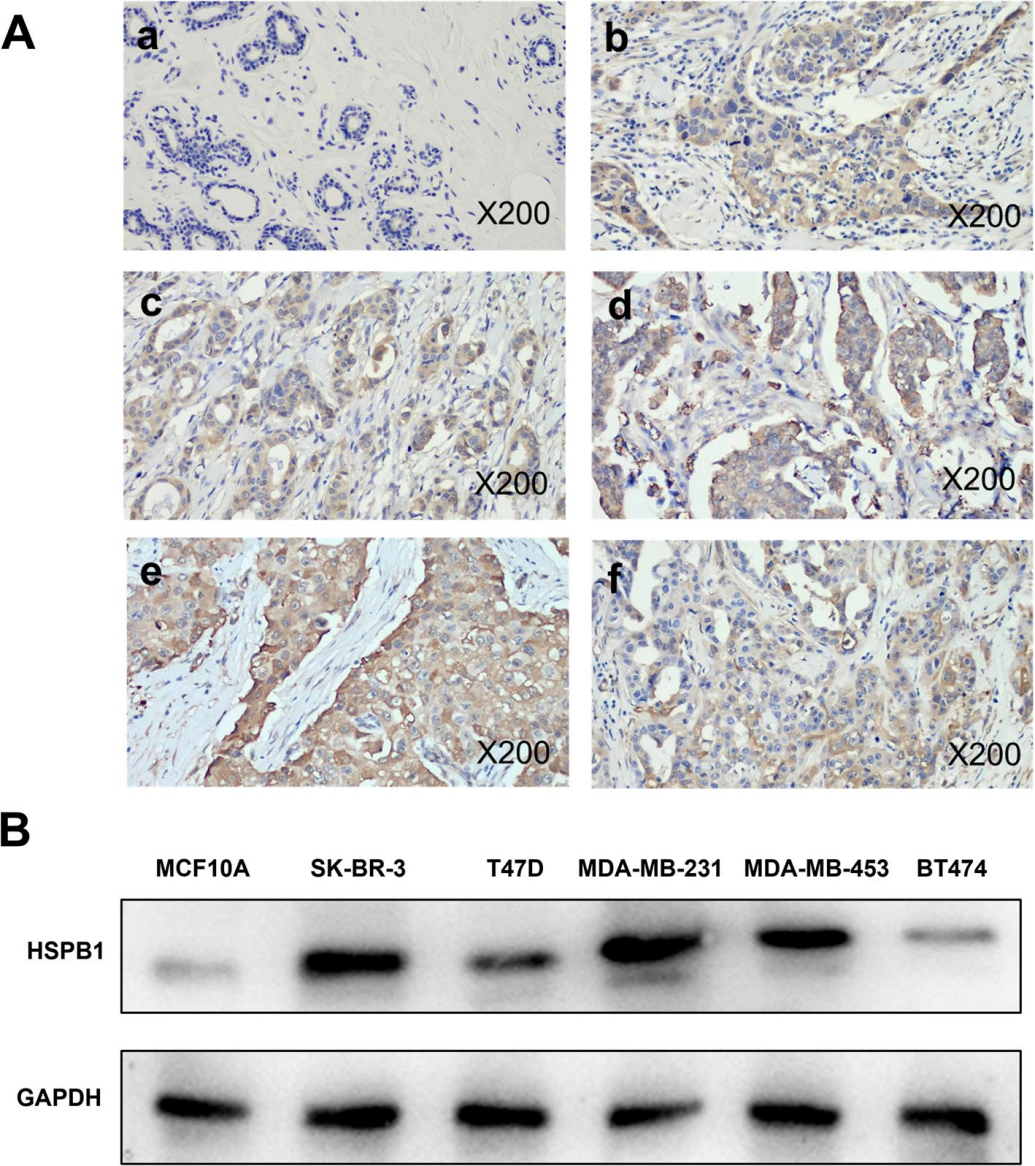
HSPB1 expression in breast cancer tissues and cells. **A** Immunohistochemical analysis of HSPB1 expression in breast tumor tissues (b-f) and normal tissues (a) (200 × magnification,). **B** Western blots showing the expression of HSPB1 protein in six breast cancer cell lines. The blots were cut prior to hybridisation with antibodies during blotting

A higher expression of HSPB1 in breast cancer tissue when comparing the immunohistochemical analysis in of 18 cancerous and non-cancerous sample pairs (Fig. 1A). In addition, *HSPB1* expression was increased in the breast cancer cell lines SK-BR-3, T47D, MDA-MB-231, MDA-MB-453, and BT474, especially in SK-BR-3 and MDA-MB-231 cells, compared with that in normal breast MCF10A cells (Fig. 1B). The findings collectively support the hypothesis that *HSPB1* is highly expressed in patients with breast cancer.

### *HSPB1* expression and clinical variables of patients with breast cancer

**Fig. 2:**
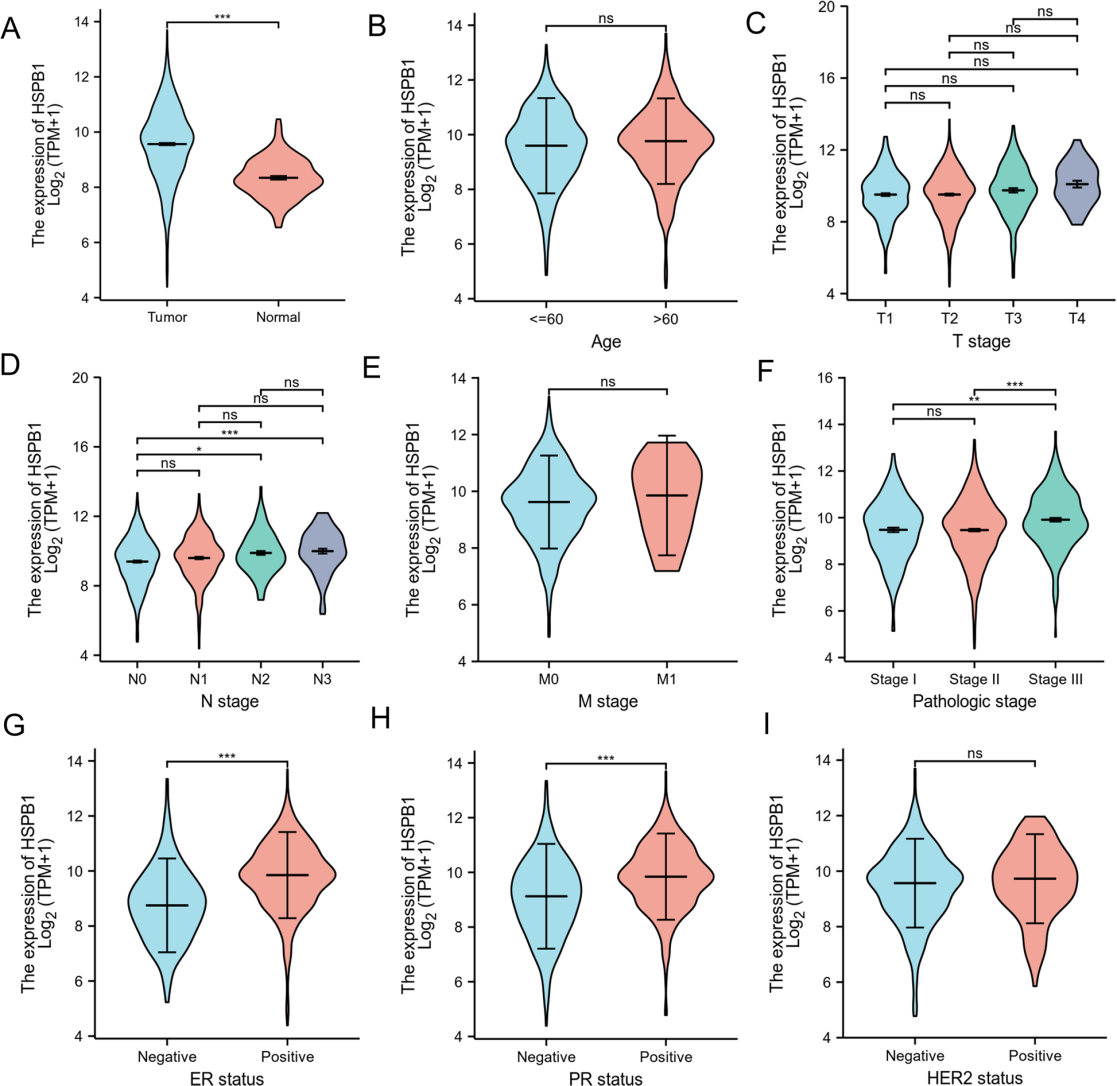
Association between the *HSPB1* expression levels and clinical characteristics in patients with breast cancer. The relationship between *HSPB1* expression and clinical characteristics, including the patient **A** status (tumor or normal), **B** age (≤ 60 and > 60), **C** T stage (T1, T2, T3, or T4), **D** N stage (N0, N1, N2, or N3), **E** M stage (M0 or M1), **F** pathologic stage (stage I, stage II, or stage III), **G** estrogen receptor (ER; negative or positive), **H** progesterone receptor (PR; negative or positive). **I** human epidermal growth factor receptor 2 (HER2; negative or positive)

To better understand the relevance and underlying mechanisms of *HSPB1* expression in breast cancer, we summarized the distribution of clinicopathological information of patients in *HSPB1* high expression group and *HSPB1* low expression group (Table 1). The data showed that the distribution of breast cancer patients in the high expression group and the low expression group was significantly different in N stage (*p* = 0.003), pathologic stage (*p* = 0.032), ER status (*p* < 0.001), and PR status (p < 0.001), but there was no significant difference between *HSPB1* expression and age, T stage, M stage, and HER2 status (All *p* > 0.05). We further examined the relationship between *HSPB1* expression and clinical characteristics, including the patient status (tumor or normal) (Fig. 2A), age (≤ 60 and > 60) (Fig. 2B), T stage (T1, T2, T3, or T4) (Fig. 2C), N stage (N0, N1, N2, or N3) (Fig. 2 D), M stage (M0 or M1) (Fig. 2E), pathologic stage (stage I, stage II, or stage III) (Fig. 2F), ER (negative or positive) (Fig. 2G), PR (negative or positive) (Fig. 2H). HER2 (negativeo or psitive) (Fig. 2I). The results showed the analysis of the pathologic stages showed that *HSPB1* expression significantly increased in stages II and III compared with stage I (*p* < 0.001). Additionally, based on ER, PR, and HER2 expression, we observed that *HSPB1* expression was significantly higher in receptor-positive samples than in receptor-negative samples (ER: *p* < 0.001; PR: *p* < 0.001).

**Table 1:** The relationship between the high and low expression of *HSPB1* and different clinical indicators in patients with breast cancer

Characteristic	Low expression of *HSPB1*	High expression of *HSPB1*	p
n	541	542	
Age, n (%)			0.133
< = 60	313 (28.9%)	288 (26.6%)	
> 60	228 (21.1%)	254 (23.5%)	
T stage, n (%)			0.409
T1	143 (13.2%)	134 (12.4%)	
T2	321 (29.7%)	308 (28.5%)	
T3	62 (5.7%)	77 (7.1%)	
T4	15 (1.4%)	20 (1.9%)	
N stage, n (%)			**0.003**
N0	282 (26.5%)	232 (21.8%)	
N1	175 (16.4%)	183 (17.2%)	
N2	52 (4.9%)	64 (6%)	
N3	26 (2.4%)	50 (4.7%)	
M stage, n (%)			0.403
M0	469 (50.9%)	433 (47%)	
M1	8 (0.9%)	12 (1.3%)	
Pathologic stage, n (%)			**0.032**
Stage I	91 (8.6%)	90 (8.5%)	
Stage II	327 (30.8%)	292 (27.5%)	
Stage III	102 (9.6%)	140 (13.2%)	
Stage IV	7 (0.7%)	11 (1%)	
ER status, n (%)			** < 0.001**
Negative	187 (18.1%)	53 (5.1%)	
Positive	330 (31.9%)	463 (44.7%)	
PR status, n (%)			** < 0.001**
Negative	225 (21.8%)	117 (11.3%)	
Positive	290 (28%)	398 (38.5%)	
HER2 status, n (%)			0.337
Negative	295 (40.6%)	263 (36.2%)	
Positive	78 (10.7%)	79 (10.9%)	

The *p*-values indicate significant differences between the low and the high expression of *HSPB1* in clinical variables. For age, median (interquartile range), and other clinical variables, the Wilcoxon rank sum test and the chi-square test were used to calculate the *p*-values, respectively

TMN stage was according to the seventh edition of the Guidelines for the American Journal of Critical Care. ER estrogen receptor, PR progesterone receptor, HER2 human epidermal growth factor receptor. Bold values indicate that *p* < 0.05

### Validation of the prognostic value of *HSPB1* in patients with breast cancer

**Fig. 3:**
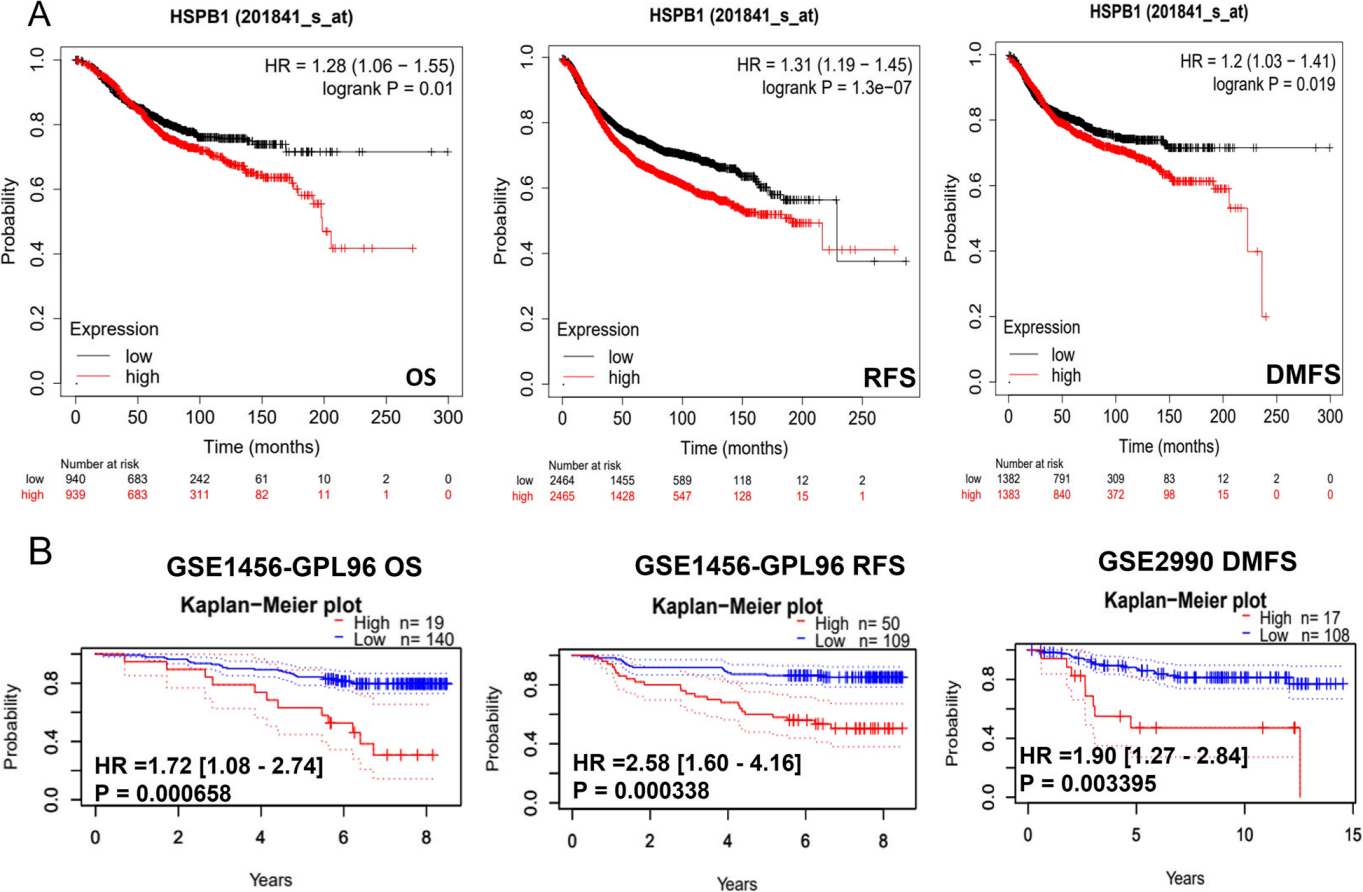
An analysis of the prognostic value of *HSPB1*. **A** Survival curves for overall survival (OS), relapse Free Survival (RFS), and distant metastasis free survival (DMFS) using the Kaplan –Meier plotter. **B** Survival curves for OS, RFS, and DMFS using the PrognoScan database

As *HSPB1* expression levels are intimately related to breast cancer progression, Kaplan–Meier survival curves were used to compare the expression levels of *HSPB1* with prognosis (Fig. 3A). The expression of *HSPB1* is significantly correlated with poor prognosis (OS: HR = 1.28, 95% CI: 1.06–1.55, *p* = 0.01; RFS: HR = 1.31, 95% CI: 1.19–1.45, *p* = 1.3e-07; DMFS: HR = 1.2, 95% CI: 1.03–1.41, *p* = 0.019). A prognostic database, PrognoScan, was employed to test the clinical outcome effect of *HSPB1*. As shown in Fig. 3B, in the GSE1456-GPL96 and GSE2990 cohorts, high-*HSPB1* were significantly worse than low-*HSPB1* on OS, RFS, and DMFS.

Logistic regression analysis (Table 2) showed a strong relationship between *HSPB1* expression and N stage (N1&N2&N3 vs. N0; OR = 1.427, 95% CI: 1.121–1.818, *p* = 0.004), pathologic stage (Stage III &Stage IV vs. Stage I & Stage II; OR = 1.516, 95% CI: 1.144–2.014, *p* = 0.004), ER status (Positive vs. Negative; OR = 4.950, 95% CI: 3.561–6.982, *p* < 0.001), and PR status (Positive vs. Negative; OR = 2.639, 95% CI: 2.019–3.464, *p* < 0.001), but there was no significant difference between *HSPB1* expression and age, T stage, M stage, HER2 status, and radiation_therapy (All *p* > 0.05).

**Table 2:** Clinical pathological features related with *HSPB1* expression according to logistic regression analysis

Characteristics	Total(N)	Odds Ratio(OR)	*P* value
Age (> 60 vs. < = 60)	1,083	1.211 (0.953–1.540)	0.118
T stage (T3&T4 vs. T1&T2)	1,080	1.322 (0.955–1.836)	0.093
N stage (N1&N2&N3 vs. N0)	1,064	1.427 (1.121–1.818)	**0.004**
M stage (M1 vs. M0)	922	1.625 (0.666–4.182)	0.293
Pathologic stage (Stage III&Stage IV vs. Stage I&Stage II)	1,060	1.516 (1.144–2.014)	**0.004**
ER status (Positive vs. Negative)	1,033	4.950 (3.561–6.982)	** < 0.001**
PR status (Positive vs. Negative)	1,030	2.639 (2.019–3.464)	** < 0.001**
HER2 status (Positive vs. Negative)	715	1.136 (0.797–1.620)	0.480
radiation_therapy (Yes vs. No)	987	1.171 (0.910–1.506)	0.219

TMN stage was according to the seventh edition of the Guidelines for the American Journal of Critical Care. ER estrogen receptor, PR progesterone receptor, HER2 human epidermal growth factor receptor. Bold values indicate that *p* < 0.05

We conducted univariate and multivariate Cox regression analyses of OS, DSS, PFI, RFS, and DMFS to investigate the relationship between prognostic factors and clinical outcomes (Tables 3, 4, 5, 6, and 7 respectively). As summarized in Table 3, in univariate Cox regression analysis we found that age (> 60 years), advanced T, N, M, and advanced pathological stages were all significantly associated with poor OS. However, patients who received radiation_therapy were significantly associated with better OS (*p* = 0.004). In multivariable Cox regression analysis, age and M stage were independent predictors of OS. Significant difference in *HSPB1* expression observed in univariate Cox regression (*p* = 0.037), but there was no significant difference in multivariate analysis of factors potentially predictive of OS.

**Table 3:** Univariate and Multivariable analysis of factors potentially predictive of overall survival

Characteristics	Total(N)	Univariate analysis		Multivariate analysis	
		Hazard ratio (95% CI)	*P* value	Hazard ratio (95% CI)	*P* value
Age	1082				
< = 60	601	Reference			
> 60	481	2.020 (1.465–2.784)	** < 0.001**	3.430 (1.821–6.460)	** < 0.001**
T stage	1079				
T1	276	Reference			
T2&T3&T4	803	1.482 (1.007–2.182)	**0.046**	1.014 (0.371–2.776)	0.978
N stage	1063				
N0	514	Reference			
N1&N2&N3	549	2.239 (1.567–3.199)	** < 0.001**	1.878 (0.885–3.985)	0.101
M stage	922				
M0	902	Reference			
M1	20	4.254 (2.468–7.334)	** < 0.001**	5.195 (1.718–15.708)	**0.004**
Pathologic stage	1059				
Stage I	180	Reference			
Stage II&Stage III&Stage IV	879	2.210 (1.313–3.721)	**0.003**	2.120 (0.473–9.507)	0.326
ER status	1032				
Negative	240	Reference			
Positive	792	0.712 (0.495–1.023)	0.066	0.572 (0.229–1.425)	0.230
PR status	1029				
Negative	342	Reference			
Positive	687	0.732 (0.523–1.024)	0.068	0.728 (0.309–1.716)	0.468
HER2 status	715				
Negative	558	Reference			
Positive	157	1.593 (0.973–2.609)	0.064	0.678 (0.317–1.450)	0.316
radiation_therapy	986				
No	434	Reference			
Yes	552	0.576 (0.394–0.841)	**0.004**	0.653 (0.354–1.203)	0.172
*HSPB1*	1082				
Low	541	Reference			
High	541	1.208 (1.074–1.455)	**0.037**		

TMN stage was according to the seventh edition of the Guidelines for the American Journal of Critical Care. ER estrogen receptor, PR progesterone receptor, HER2 human epidermal growth factor receptor. Bold values indicate that *p* < 0.05

**Table 4:** Univariate and Multivariable analysis of factors potentially predictive of disease specific survival

Characteristics	Total(N)	Univariate analysis		Multivariate analysis	
		Hazard ratio (95% CI)	*P* value	Hazard ratio (95% CI)	*P* value
Age	1062				
< = 60	590	Reference			
> 60	472	1.445 (0.941–2.219)	0.093	1.280 (0.751–2.180)	0.364
T stage	1059				
T1	274	Reference			
T2&T3&T4	785	1.781 (1.033–3.071)	**0.038**	1.271 (0.525–3.080)	0.595
N stage	1044				
N0	511	Reference			
N1&N2&N3	533	3.797 (2.222–6.489)	** < 0.001**	2.840 (1.451–5.559)	**0.002**
M stage	903				
M0	884	Reference			
M1	19	7.454 (3.988–13.931)	** < 0.001**	6.663 (3.139–14.142)	** < 0.001**
Pathologic stage	1041				
Stage I	178	Reference			
Stage II&Stage III&Stage IV	863	3.396 (1.478–7.803)	**0.004**	1.118 (0.300–4.165)	0.869
ER status	1013				
Negative	232	Reference			
Positive	781	0.559 (0.351–0.891)	**0.015**	0.435 (0.193–0.978)	**0.044**
PR status	1010				
Negative	334	Reference			
Positive	676	0.519 (0.334–0.807)	**0.004**	0.707 (0.321–1.555)	0.388
HER2 status	704				
Negative	550	Reference			
Positive	154	1.477 (0.740–2.948)	0.269		
radiation_therapy	977				
No	430	Reference			
Yes	547	0.791 (0.483–1.295)	0.351		
*HSPB1*	1062				
Low	528	Reference			
High	534	1.515 (1.239–1.837)	**0.029**		

TMN stage was according to the seventh edition of the Guidelines for the American Journal of Critical Care. ER estrogen receptor, PR progesterone receptor, HER2 human epidermal growth factor receptor. Bold values indicate that *p* < 0.05

**Table 5:** Univariate and Multivariable analysis of factors potentially predictive of progress free interval

Characteristics	Total(N)	Univariate analysis		Multivariate analysis	
		Hazard ratio (95% CI)	*P* value	Hazard ratio (95% CI)	*P* value
Age	1082				
< = 60	601	Reference			
> 60	481	1.253 (0.904–1.738)	0.175		
T stage	1079				
T1	276	Reference			
T2&T3&T4	803	1.886 (1.241–2.867)	**0.003**	1.609 (0.731–3.542)	0.237
N stage	1063				
N0	514	Reference			
N1&N2&N3	549	2.333 (1.621–3.357)	** < 0.001**	1.771 (1.123–2.794)	**0.014**
M stage	922				
M0	902	Reference			
M1	20	8.315 (4.829–14.315)	** < 0.001**	6.005 (3.115–11.573)	** < 0.001**
Pathologic stage	1059				
Stage I	180	Reference			
Stage II&Stage III&Stage IV	879	2.268 (1.325–3.880)	**0.003**	0.867 (0.316–2.381)	0.782
ER status	1032				
Negative	240	Reference			
Positive	792	0.622 (0.436–0.887)	**0.009**	0.700 (0.394–1.244)	0.224
PR status	1029				
Negative	342	Reference			
Positive	687	0.558 (0.400–0.779)	** < 0.001**	0.593 (0.343–1.025)	0.061
HER2 status	715				
Negative	558	Reference			
Positive	157	1.228 (0.712–2.119)	0.461		
radiation_therapy	986				
No	434	Reference			
Yes	552	0.899 (0.631–1.281)	0.555		
*HSPB1*	1082				
Low	541	Reference			
High	541	1.425 (1.137–1.750)	**0.041**		

TMN stage was according to the seventh edition of the Guidelines for the American Journal of Critical Care. ER estrogen receptor, PR progesterone receptor, HER2 human epidermal growth factor receptor. Bold values indicate that *p* < 0.05

**Table 6:** Univariate and Multivariable analysis of factors potentially predictive of relapse-free survival

Characteristics	Total(N)	Univariate analysis		Multivariate analysis	
		Hazard ratio (95% CI)	*P* value	Hazard ratio (95% CI)	*P* value
Age	4929				
< = 60	2464	Reference			
> 60	2465	1.125 (0.832–2.056)	0.127	1.245(0.796–2.214)	0.414
T stage	4920				
T1	1274	Reference			
T2&T3&T4	3646	1.315 (1.007–2.731)	0.052	1.373 (0.826–3.134)	0.125
N stage	4894				
N0	2143	Reference			
N1&N2&N3	2751	2.215 (2.222–5.194)	** < 0.001**	2.853 (1.321–5.317)	**0.013**
M stage	4801				
M0	4637	Reference			
M1	164	6.153 (3.064–12.612)	** < 0.001**	5.132 (3.428–13.241)	** < 0.001**
Pathologic stage	4887				
Stage I	1035	Reference			
Stage II&Stage III&Stage IV	3852	3.491 (1.613–7.423)	**0.007**	1.307 (0.403–3.459)	0.749
ER status	4872				
Negative	938	Reference			
Positive	3934	0.459 (0.313–0.725)	**0.003**	0.413 (0.213–0.728)	**0.045**
PR status	4860				
Negative	1104	Reference			
Positive	3756	0.617 (0.414–0.876)	**0.017**	0.834 (0.414–1.625)	0.407
HER2 status	4507				
Negative	3754	Reference			
Positive	753	1.603 (0.629–2.714)	0.315		
radiation_therapy	4736				
No	1975	Reference			
Yes	2761	0.691 (0.315–1.176)	0.453		
*HSPB1*	4929				
Low	2464	Reference			
High	2465	1.479 (1.124–1.893)	**0.038**		

TMN stage was according to the seventh edition of the Guidelines for the American Journal of Critical Care. ER estrogen receptor, PR progesterone receptor, HER2 human epidermal growth factor receptor. Bold values indicate that *p* < 0.05

**Table 7:** Univariate and Multivariable analysis of factors potentially predictive of distant metastasis-free survival

Characteristics	Total(N)	Univariate analysis		Multivariate analysis	
		Hazard ratio (95% CI)	*P* value	Hazard ratio (95% CI)	*P* value
Age	2765				
< = 60	1382	Reference			
> 60	1383	1.152 (0.831–1.625)	0.273		
T stage	2759				
T1	573	Reference			
T2&T3&T4	2186	1.974 (1.164–2.977)	**0.004**	1.527 (0.822–2.312)	0.313
N stage	2753				
N0	1374	Reference			
N1&N2&N3	1378	2.712 (1.742–3.529)	** < 0.001**	1.631 (1.042–2.636)	**0.034**
M stage	2649				
M0	1913	Reference			
M1	736	7.137 (3.785–12.465)	** < 0.001**	5.024 (3.607–10.143)	** < 0.001**
Pathologic stage	2746				
Stage I	381	Reference			
Stage II&Stage III&Stage IV	2365	3.539 (1.613–5.746)	**0.013**	1.463 (0.607–2.594)	0.815
ER status	2731				
Negative	643	Reference			
Positive	2088	0.703 (0.342–1.327)	**0.019**	0.820 (0.492–1.393)	0.314
PR status	2716				
Negative	721	Reference			
Positive	1995	0.524 (0.311–0.816)	** < 0.001**	0.573 (0.249–1.214)	0.073
HER2 status	1843				
Negative	1449	Reference			
Positive	394	1.304 (0.801–2.024)	0.526		
radiation_therapy	2679				
No	1163	Reference			
Yes	1516	0.649 (0.434–1.407)	0.434		
*HSPB1*	2765				
Low	1382	Reference			
High	1383	1.614 (1.307–1.942)	**0.045**		

TMN stage was according to the seventh edition of the Guidelines for the American Journal of Critical Care. ER estrogen receptor, *PR* progesterone receptor, *HER2* human epidermal growth factor receptor. Bold values indicate that *p* < 0.05

More advanced T, N, M, and pathologic stages were associated with worse DSS, and ER or PR positive statuses were associated with better DSS. The advanced N and M stages were independent predictors of DSS (Table 4), PFI (Table 5), RFS (Table 6), and DMFS (Table 7). Significant difference in *HSPB1* expression observed in univariate Cox regression (DSS: *p* = 0.029; PFI: *p* = 0.041; RFS: *p* = 0.038; DMFS: *p* = 0.045), but there was no significant difference in multivariate analysis of factors potentially predictive of DSS, PFI, RFS, and DMFS.

*HSPB1* expression differences observed in the univariate Cox regression and multivariate analyses were not significant.

These data suggest that patients with breast cancer with high *HSPB1* expression have a poor prognosis. *HSPB1* is not an independent marker for OS, DSS, PFI, RFS, or DMFS.

### Identification of *HSPB1*-interacting genes and proteins and pathway analysis

**Fig. 4:**
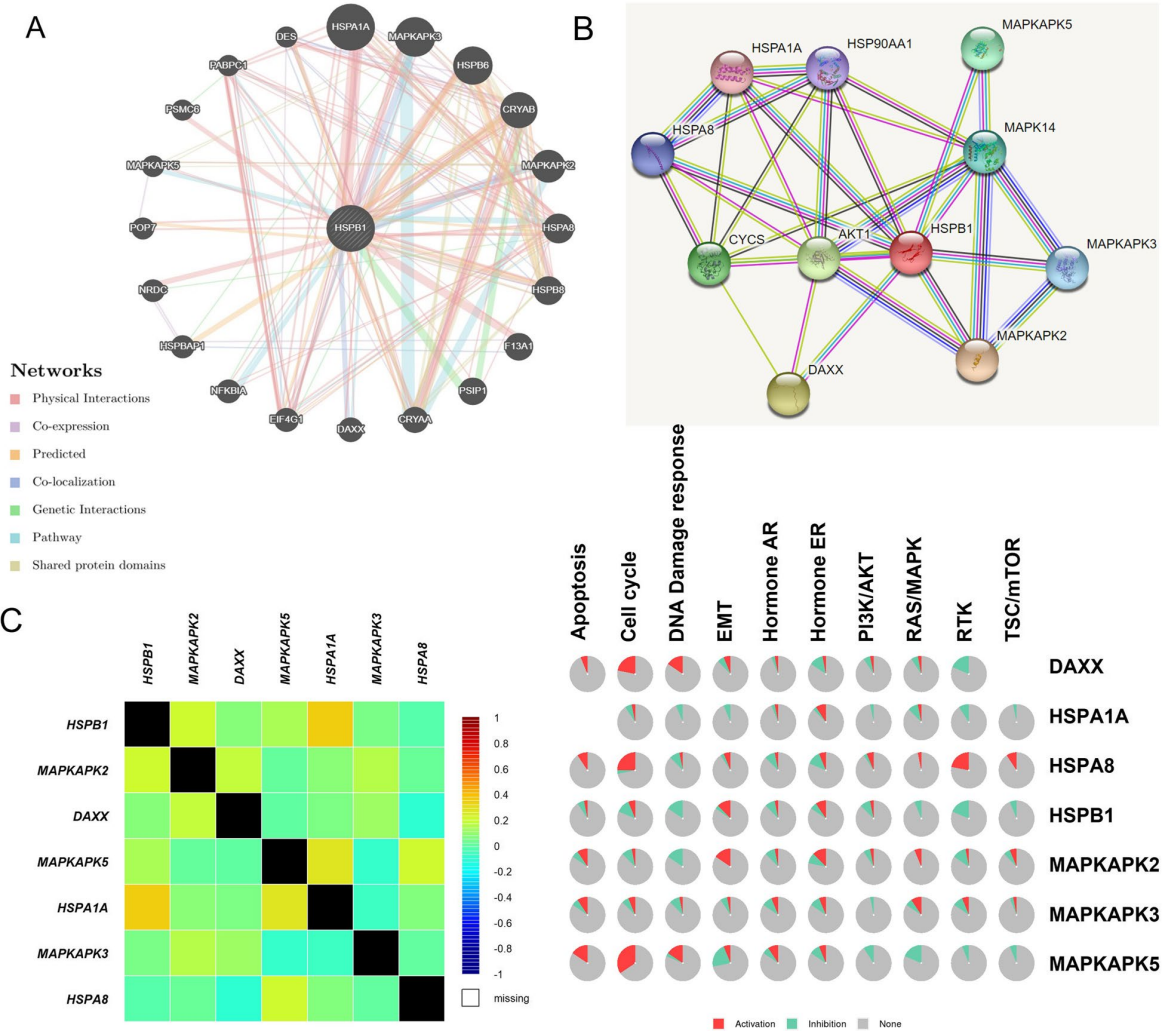
Identification of *HSPB1*-interacting genes and proteins and pathway analysis. The gene–gene interaction network and protein–protein interaction network of *HSPB1* were constructed using GeneMANIA **A** and STRING **B**. **C** The correlation between* HSPB1* and the altered neighboring genes using bc-GenExMiner v 4.8. **D** The GSCALite protocol was used to analyze the pathway activity (activation and inhibition)

We used GeneMANIA to create the gene–gene interaction network for *HSPB1* and the altered neighboring genes (Fig. 4A). We observed that the 20 most frequently altered genes were remarkably associated with *HSPB1* expression. The proteins expressed by these six genes (mitogen-activated protein kinase activated protein kinase 2 (*MAPKAPK2*), death domain-associated protein (*DAXX*), *MAPKAPK5*, heat shock protein family A member 1A (*HSPA1A*), *MAPKAPK3*, and heat shock protein family A member 8 (*HSPA8*)) were found to interact with *HSPB1* in the STRING database, with correlation scores of 0.998, 0.996, 0.992, 0.964, 0.981, and 0.972, respectively (Fig. 4B). We then used bc-GenExMinerv 4.8 to confirm the relationship between *HSPB1* and the six genes (Fig. 4C). *HSPB1* expression was positively associated with *HSPA1A* (*r* = 0.35, *p* < 0.0001), and moderate associated with *MAPKAPK2* (*r* = 0.200, *p* < 0.0001). Our study used the GSCALite database to analyze pathways of altered neighboring genes (Fig. 4D). These results indicated that *HSPB1* expression activated the EMT. Moreover, we observed that *DAXX*, *HSPA8*, and *MAPKAPK5* mainly activate the cell cycle. However, the expression of *HSPB1* mainly inhibited DNA damage response, and the Ras/MAPK, and RTK. These findings suggested that *HSPB1* was associated with the occurrence, progression, and metastasis of breast cancer.

### Transient knockdown of *HSPB1* inhibited proliferation in breast cancer cells

**Fig. 5:**
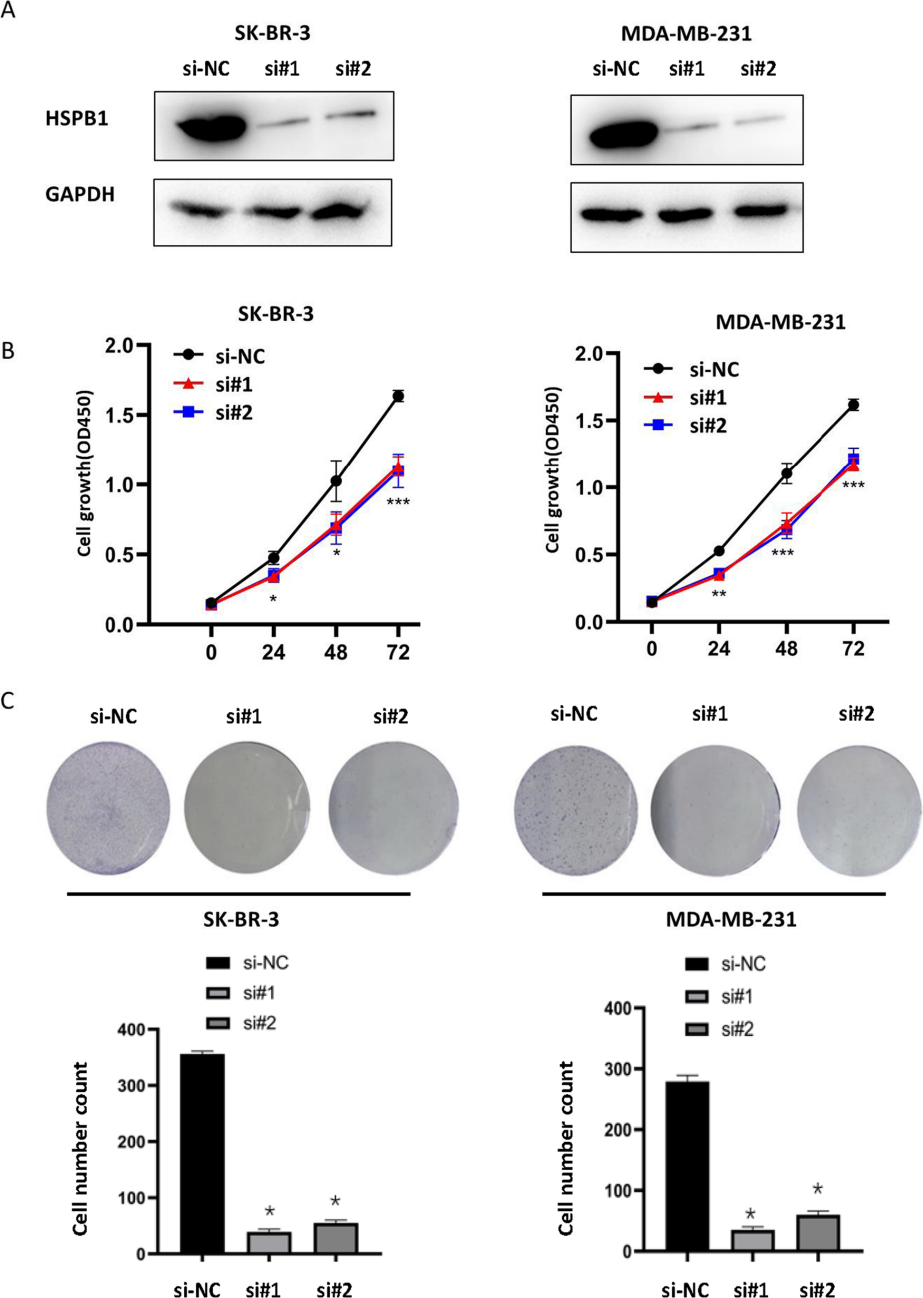
Transient knockdown of *HSPB1* affects breast cancer cell proliferation. **A** Verification of *HSPB1* expression in SK-BR-3 and MDA-MB-231 cell lines via western blot. Growth curves **B** and Colony-forming efficiency **C** in SK-BR-3 and MDA-MB-231 cells before and after *HSPB1* transient knockdown. The quantification of each analysis is shown in the following figure. All assays were performed in triplicate. The data are presented as means ± SEM. (*p < 0.05; **p < 0.01; *** *p* < 0.001). The blots were cut prior to hybridisation with antibodies during blotting

In this study, SK-BR-3 and MDA-MB-231 breast cancer cells with the high protein expression of *HSPB1* were selected as research subjects. Two siRNAs targeting *HSPB1* were transfected into SK-BR-3 and MDA-MB-231 cells to knock down *HSPB1*. These results suggested that *HSPB1* was successfully knocked down (Fig. 5A). Additionally, we tested the ability of *HSPB1* to promote cell growth by using CCK8 and colony formation assays (Fig. 5B and C). Cell proliferation was significantly decreased in SK-BR-3 and MDA-MB-231 cells after transient *HSPB1* knockdown compared with that in the si-NC group. Moreover, the transient knockdown lines exhibited significantly inhibited cell colony-forming numbers compared to the si-NC group.

### *HSPB1* transient knockdown inhibited cell migration/invasion and promoted cell apoptosis in breast cancer cells

**Fig. 6:**
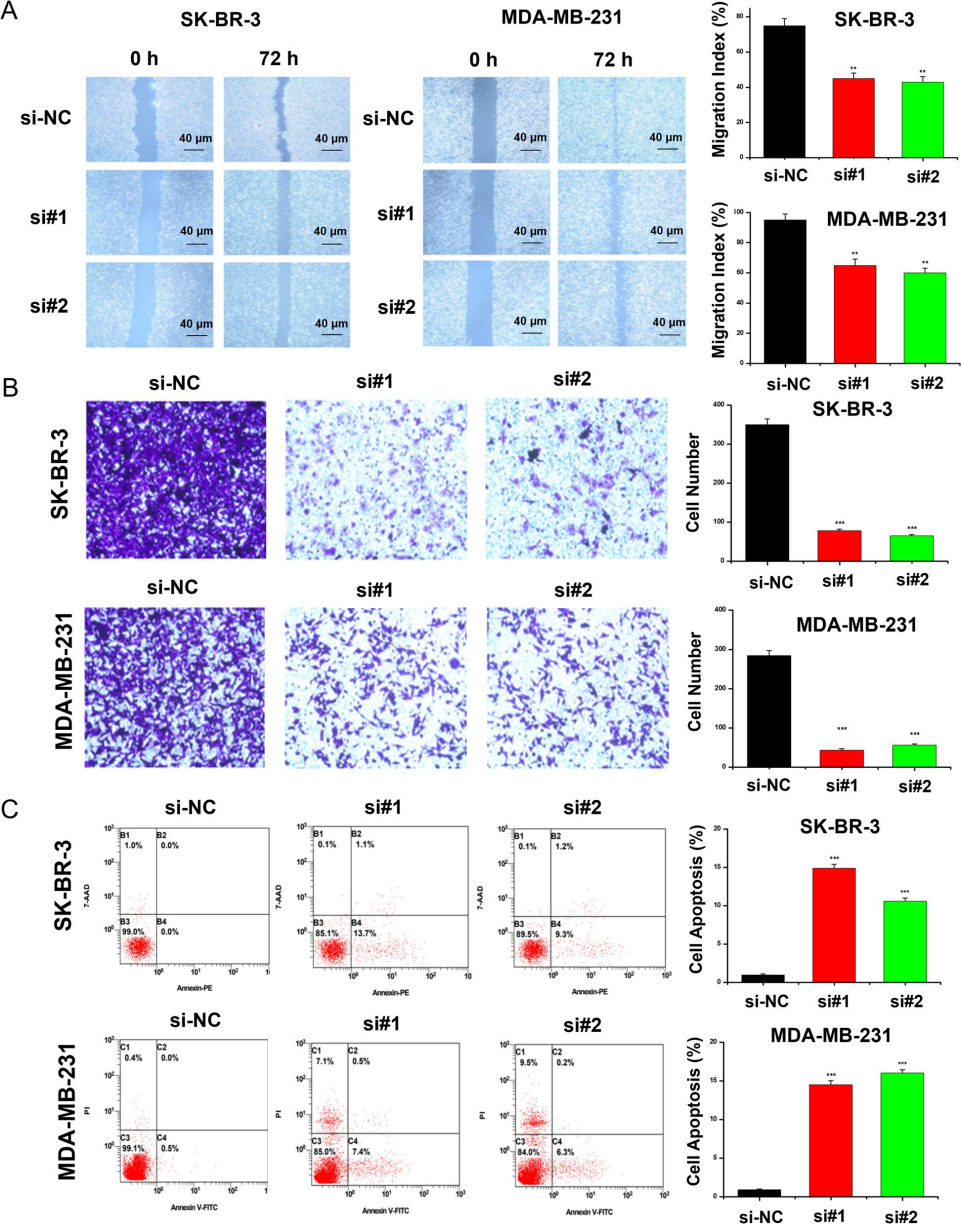
Transient knockdown of *HSPB1* affects breast cancer cell migration, invasion, and apoptosis. **A** The effect of transient knockdown of *HSPB1* expression on the cell migration was determined by using the wound healing assay. The quantification of each analysis is shown in the right figure. The experiments were carried out in triplicate (**p < 0.01). The scratch area was calculated using Image J software. Cell scratch area (0 h) minus cell scratch area (72 h) to get the cell migration area, the percentage of cell migration area to cell scratch area (0 h) is the cell migration index. **B** Transwell® assay was used to determine the cell invasion after *HSPB1* transient knockdown. The quantification of each analysis is shown in the right figure. The experiments were carried out in triplicate (***p < 0.001). **C** The impact of transient knockdown *HSPB1* expression on cellular apoptosis as determined via flow cytometry. All assays were performed in triplicate. SK-BR-3 and MDA-MB-231 breast cancer cell lines were used in this study. The right picture shows the percentage of cell apoptosis. The experiments were carried out in triplicate (***p < 0.001)

Cell invasion ability of SK-BR-3 and MDA-MB-231 cells was assessed at 0 and 72 h after injury using a wound-healing assay. Our data showed that in the scratch experiment, the wound healing rate of *HSPB1* knockdown treated cells was significantly lower than that of control cells, and their migration ability was significantly reduced (Fig. 6A). Additionally, Transwell® inserts were was used to evaluate cell migration (Fig. 6B). The number of migrated SK-BR-3 and MDA-MB-231 cells after *HSPB1* transient knockdown was lower than that of the si-NC group. Next, we studied the effect of *HSPB1* on breast cancer cell apoptosis using flow cytometry. *HSPB1* transient knockdown promoted apoptosis in SK-BR-3 and MDA-MB-231 cells (Fig. 6C). These findings support the hypothesis that *HSPB1* affects the migration, invasion, and apoptosis in breast cancer.

### *HSPB1* might be involved in the metastasis of breast cancer

**Fig. 7:**
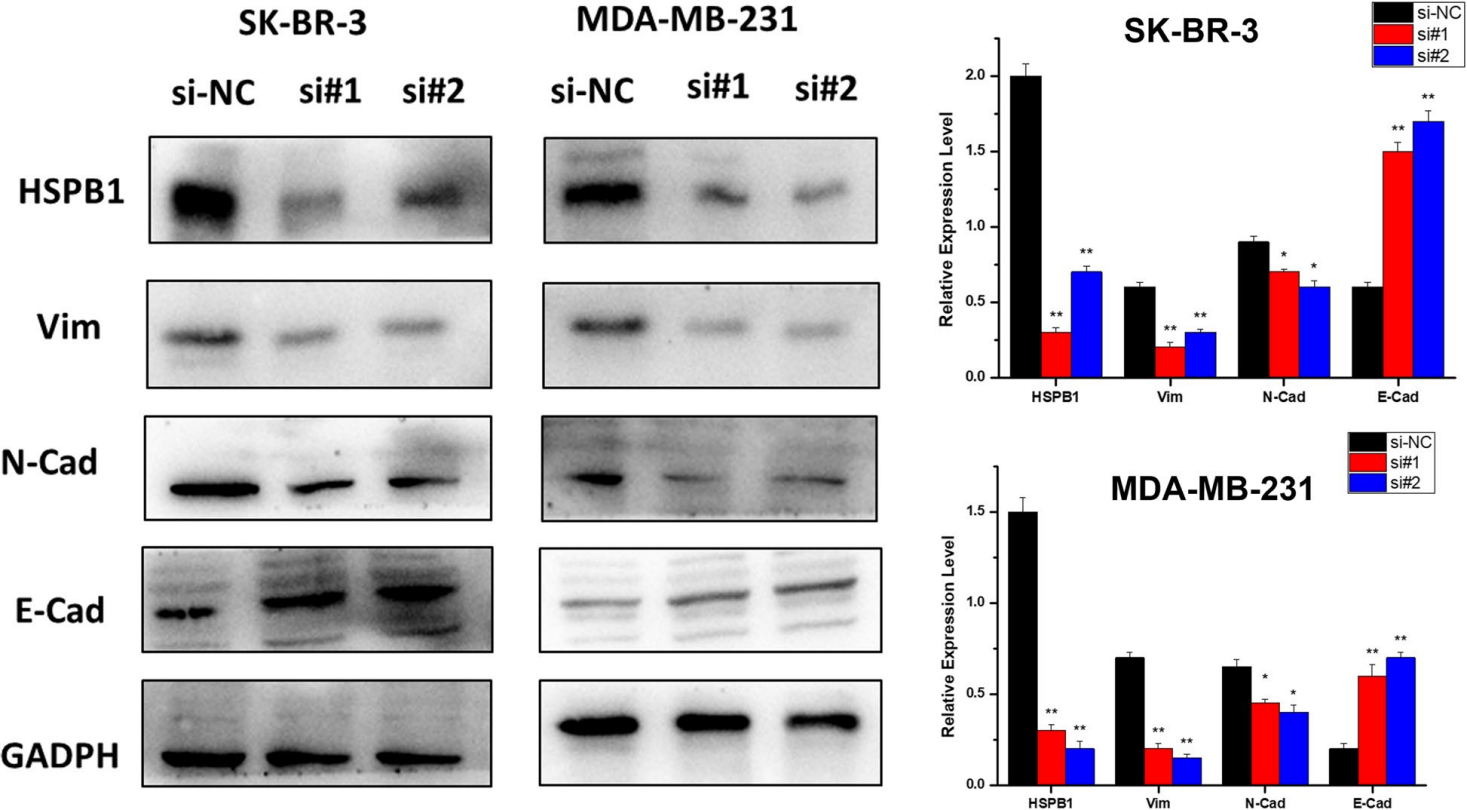
Transient knockdown of *HSPB1* affects the cell epithelial-mesenchymal transition (EMT) process. **A** Vimentin, N-cadherin, and E-cadherin expression levels after si*HSPB1*-treatment as measured using western blotting. All assays were performed in triplicate. The blots were cut prior to hybridisation with antibodies during blotting. The quantification of each analysis is shown in the right figure. The experiments were carried out in triplicate (*p < 0.05; **p < 0.01)

**Fig. 8:**
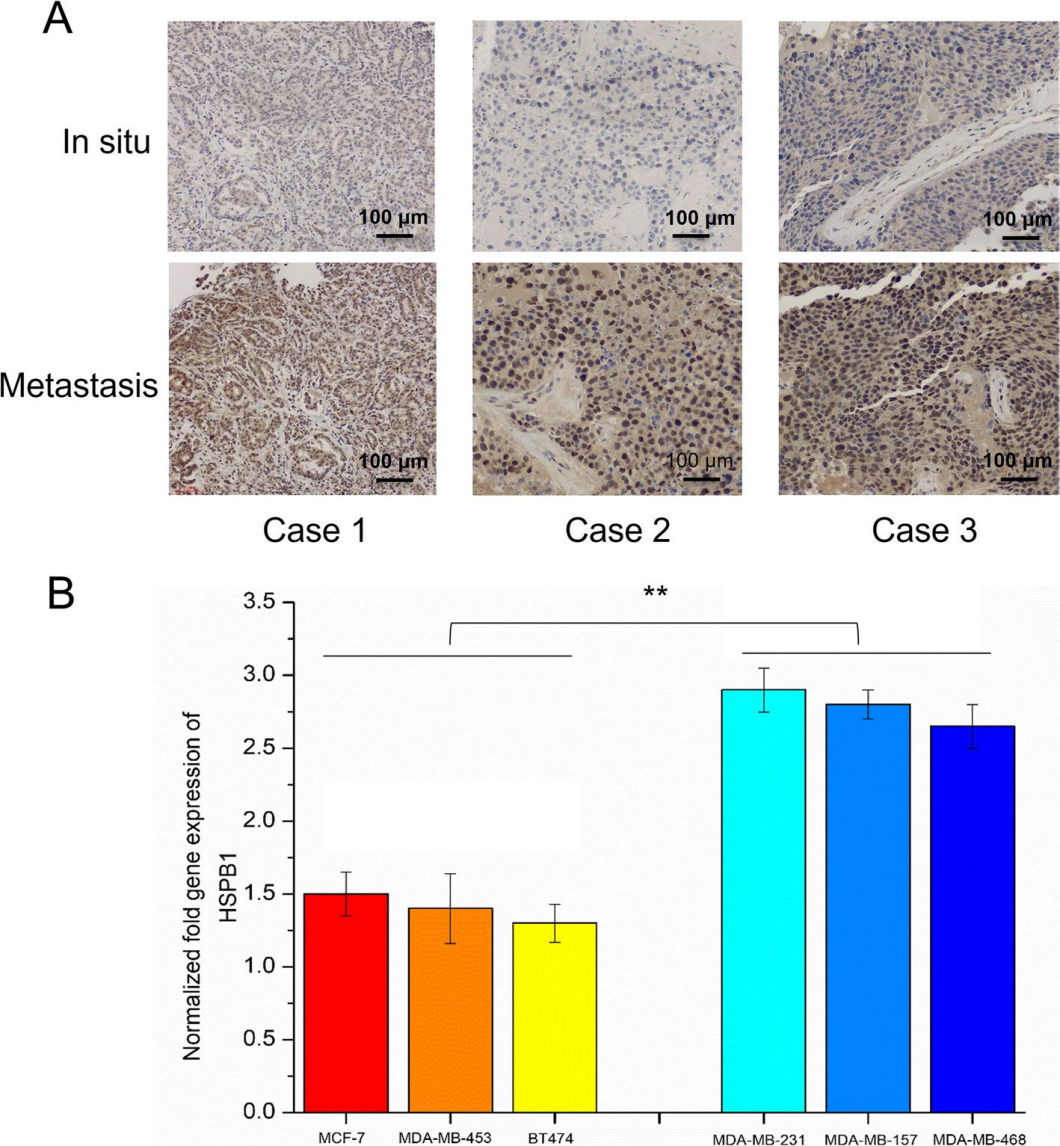
**A** Immunohistochemical staining showing the expression of HSPB1 in situ and in breast cancer metastasis. **B** The expression of *HSPB1* in three triple-negative breast cancer cell lines (MDA-MB-468, MDA-MB-157, and MDA-MB-231) and three other breast cancer cell lines (MCF-7, MDA-MB-453, and BT474) were measured through quantitative real-time PCR (** *p* < 0.01)

To evaluate the effect of *HSPB1* on EMT in breast cancer cells. The expression of E-cadherin, N-cadherin, and vimentin were analyzed after *HSPB1* transient knockdown. We discovered that *HSPB1* knockdown significantly reduced the expression of vimentin and N-cadherin, but E-cadherin expression was significantly increased (Fig. 7). We hypothesized that *HSPB1* is involved in breast cancer metastasis. To explore this hypothesis, *HSPB1* expression was first analyzed in the metastasis and in situ groups of with breast cancer. *HSPB1* was highly expressed in patients with metastatic breast cancer, but low in those with carcinoma in situ (Fig. 8A). We then used RT-PCR to detect the expression of *HSPB1* in triple-negative breast cancer cells (MDA-MB-468, MDA-MB-157, and MDA-MB-231) and non-triple-negative breast cancer cells (MCF-7, MDA-MB-453, and BT474). Concordantly, *HSPB1* expression was significantly increased in triple-negative breast cancer cells (*p* < 0.01) (Fig. 8B). Overall, *HSPB1* may be involved in breast cancer metastasis.

## Discussion

Despite continuous improvements in breast cancer research and treatment methods, the incidence rate continues to increase. Therefore, there is a pressing need to explore the mechanisms leading to breast cancer metastasis. *HSPB1* is widely expressed in various tumors [7, 10, 27] and may contribute to tumor proliferation, migration, and drug resistance [28, 29]. Our study evaluated the expression levels, clinicopathological associations, clinical significance, and influence on metastasis of *HSPB1* in breast cancer.

Many studies have investigated *HSPB1* in relation to various cancer types. Huang et al. (2010) reported that *HSPB1* was overexpressed in gastric adenocarcinoma tissue and that serum levels of *HSPB1* were increased in patients with gastric adenocarcinoma, which may indicate gastric malignancy and thus its detection may be helpful for screening gastric adenocarcinoma [30]. Another study suggested that the expression of *HSPB1* may be used to predict poor prognosis and transfer tendency in prostate cancer and demonstrated that *HSPB1* was promoted in an insulin-like growth factor 1-dependent manner [31]. They also reported that the phosphorylation of extracellular signal-regulated kinase 1 and Akt stabilizes the BAD /14–3-3 protein complex, reducing the rate of prostate cancer cell apoptosis. In addition, *HSPB1* expression in prostate cancer cells is significantly increased after androgen deprivation and chemotherapy and acts as a molecular chaperone for cell protection, making cells resistant to drugs [32]. These results indicated that *HSPB1* could be used as a target for radiotherapy sensitization in prostate cancer. Furthermore, *HSPB1* antibody levels are elevated in patients with breast cancer [33]. Despite these observations, a comprehensive study of *HSPB1* in breast cancer has not yet been conducted, and it remains unclear how *HSPB1* affects breast cancer occurrence and development [34]. According to our findings, the expression of *HSPB1* was considerably upregulated in breast cancer tissues, which is consistent with a previous report [35]. Notably, we also confirmed that *HSPB1* expression was associated with the clinical features of patients with breast cancer. Higher *HSPB1* expression was closely correlated with pathologic stage, ER, and PR. The increased expression of *HSPB1* observed in late-stage malignancies suggests that *HSPB1* may contribute to cancer development. Therefore, we propose *HSPB1* as a marker of poor survival in patients with breast cancer.

To evaluate the prognostic potential of *HSPB1* in breast cancer, we used the Kaplan–Meier survival curve to analyze the effect of *HSPB1* expression level on survival. Patients with higher *HSPB1* level had remarkably worse OS, RFS, and DMFS. Consistent results were obtained using the online tool PrognoScan. However, *HSPB1* expression differences were not significant in univariate logistic regression and multivariate analyses for OS, DSS, PFI, RFS, and DMFS. We confirmed that *HSPB1* is not an independent marker for OS, DSS, PFI, RFS, or DMFS. According to these findings, *HSPB1* may be a prognostic biomarker for breast cancer and may facilitate the development of targeted precision oncology.

Apoptosis plays a vital role in cancer [36]. *HSPB1* directly inhibits the activation of caspases to inhibit cell apoptosis [37], prevent multiple apoptotic effects from inducing cell death, and regulate apoptosis signaling pathway, [38]. In this study, a transient knockdown of *HSPB1* inhibited cell proliferation and migration/invasion activity and promoted apoptosis of breast cancer cells. EMT is the most crucial pathway for tumor cell invasion and metastasis [39–41]. During this process, cells gain the ability to move, invade, and separate from the epithelial membrane. EMT is associated with tumorigenesis, metastasis, and drug resistance [42]. Similar to Yun et al. [43], we confirmed that the transient knockdown of *HSPB1* significantly reduced the expression of vimentin, and N-cadherin, but upregulated E-cadherin expression. As an oncogene, *HSPB1* promotes the activity of breast cancer cells by regulating the EMT process, thus establishing *HSPB1* as a promising biomarker for the diagnosis and treatment of breast cancer; however, the potential mechanism by which *HSPB1* affects cell proliferation and EMT requires further investigation.

## Conclusions

Overall, high *HSPB1* expression predicted poor clinical outcomes, meaning that it holds potential as a novel prognostic biomarker for breast cancer. *HSPB1* knockdown inhibited the proliferation, migration, invasion, and apoptosis of breast cancer cells. This study advances our current understanding of the role of *HSPB1* as a prognostic marker for breast cancer treatment.

## Supplementary Information

**Additional file 1.**

**Additional file 2. **

**Additional file 3.** 

**Additional file 4.** 

**Additional file 5.** 

**Additional file 6.** 

**Additional file 7.** 

**Additional file 8.** 

**Additional file 9.** 

**Additional file 10.** 

**Additional file 11.** 

## Data Availability

The authors confirm that the data supporting the findings of this study are available within the article and its supplementary materials.

## Abbreviations

CCK8: Cell counting kit 8
DAXX: Death domain associated protein
DMFS: Distant metastasis-free survival
DSS: Disease-specific survival
EMT: Epithelial-mesenchymal transition
ER: Estrogen receptor; FBS, fetal bovine serum
HER2: Human epidermal growth factor receptor 2
HR: Hazard ratio
HSPA1A: Heat shock protein family A member 1A
HSPA8: Heat shock protein family A member 8
HSPB1: Heat shock protein beta-1
MAPKAPK: Mitogen-activated protein kinase activated protein kinase
OS: Overall survival
PBS: Phosphate buffered saline
PFI: Progression-free interval
PR: Progesterone receptor
RFS: Relapse-free survival
RT-PCR: Quantitative real-time PCR
TCGA: The Cancer Genome Atlas
