# Supplementary material for: High HSPB1 expression predicts poor clinical outcomes and correlates with breast cancer metastasis
Source: BMC Cancer. 2023 Jun 3;23:501. doi: 10.1186/s12885-023-10983-3 (PMC10239126; doi:10.1186/s12885-023-10983-3)
Supplement: Supplementary file 2 — Additional file 2. [file 12885_2023_10983_MOESM2_ESM.pdf]

Institution:	Run Date: 20-Jan-21, 16:51:36
Protocol :20210120 • ，231-1 005 NoRead 00020760 582.PRO	Sample ID: 20210120 • ，231-1
Listmode Replay: New Protocol	User ID: user1
Analysis Date: 02-Mar-2021, 17:46:41	Acquisition Time/Events: 15.6s / 15000 (PROTOCOL)
Settings File: Settings modified during acquisition, N/A	
Listmode File: 20210120 • ，231-1 005 NoRead 00020760 582.LMD	Instrument SN: AU18113 Software Version: Navios 1.1

(30000) [A] FL1 INT LOG/FL3 INT LOG

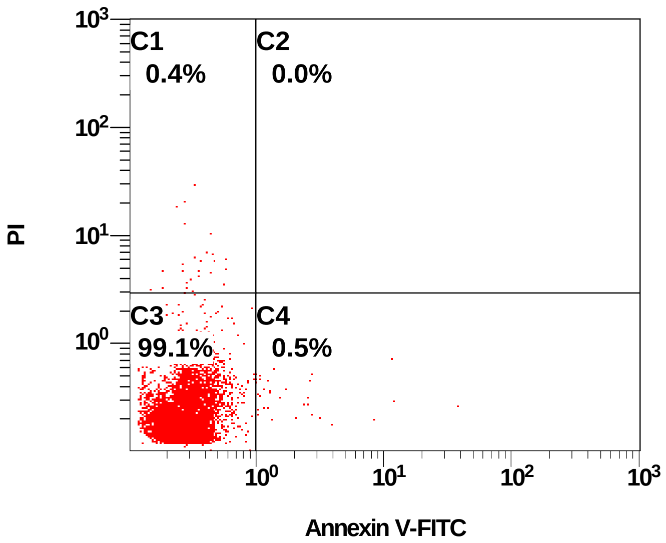

Statistical Analysis

PROGRAM INFORMATION

File:- 20210120 • , 231-1 005 NoRead 00020760 582.LMD					
Gate:- A [A]					
Compensation:-					
Region	Number	%Total	%Gated	X-Mean	Y-Mean
ALL	12883	85.89	100.00	0.299	0.286
ALL	12883	85.89	100.00	0.299	354
ALL	12883	85.89	100.00	0.286	354
C1	52	0.35	0.40	0.35	9.52
C2	0	0.00	0.00	0	0
C3	12765	85.10	99.08	0.281	0.248
C4	66	0.44	0.51	3.56	0.356

File:- 20210120 • , 231-1 005 NoRead 00020760 582.LMD					
Gate:- Ungated					
Compensation:-					
Region	Number	%Total	%Gated	X-Mean	Y-Mean
ALL	15000	100.00	100.00	435	549
A	12883	85.89	85.89	354	480
