# Supplementary material for: High HSPB1 expression predicts poor clinical outcomes and correlates with breast cancer metastasis
Source: BMC Cancer. 2023 Jun 3;23:501. doi: 10.1186/s12885-023-10983-3 (PMC10239126; doi:10.1186/s12885-023-10983-3)
Supplement: Supplementary file 3 — Additional file 3. [file 12885_2023_10983_MOESM3_ESM.pdf]

Institution:

Protocol :20210120 • ，231-2 004 NoRead 00020769 591.PRO

Listmode Replay: New Protocol

Analysis Date: 02-Mar-2021, 17:54:39

Settings File: ƒ „ .PRO, 20-Jan-2021, 16:45:06

Listmode File: 20210120 • ，231-2 004 NoRead 00020769 591.LMD

Run Date: 20-Jan-21, 17:07:32

Sample ID: 20210120 • ，231-2

User ID: user1

Acquisition Time/Events: 59.6s / 15000 (PROTOCOL)

Instrument SN: AU18113 Software Version: Navios 1.1

(30000) [A] FL1 INT LOG/FL3 INT LOG

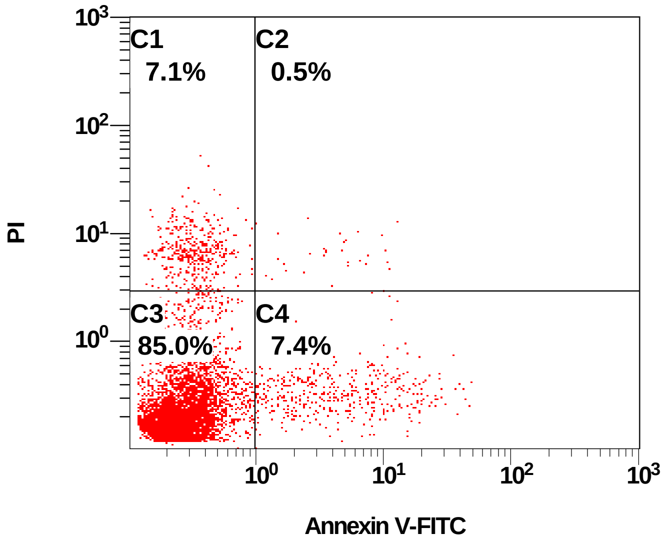

Statistical Analysis

PROGRAM INFORMATION

File:- 20210120 • , 231-2 004 NoRead 00020769 591.LMD					
Gate:- A [A]					
Compensation:-					
Region	Number	%Total	%Gated	X-Mean	Y-Mean
ALL	11590	77.27	100.00	0.833	0.871
ALL	11590	77.27	100.00	0.833	299
ALL	11590	77.27	100.00	0.871	299
C1	822	5.48	7.09	0.355	7.89
C2	60	0.40	0.52	5.15	6.56
C3	9850	65.67	84.99	0.284	0.292
C4	858	5.72	7.40	7.29	0.397

File:- 20210120 • , 231-2 004 NoRead 00020769 591.LMD					
Gate:- Ungated					
Compensation:-					
Region	Number	%Total	%Gated	X-Mean	Y-Mean
ALL	15000	100.00	100.00	456	498
A	11590	77.27	77.27	299	353
