# Supplementary material for: High HSPB1 expression predicts poor clinical outcomes and correlates with breast cancer metastasis
Source: BMC Cancer. 2023 Jun 3;23:501. doi: 10.1186/s12885-023-10983-3 (PMC10239126; doi:10.1186/s12885-023-10983-3)
Supplement: Supplementary file 4 — Additional file 4. [file 12885_2023_10983_MOESM4_ESM.pdf]

Institution:
Protocol :20210120 • ，231-2 002 NoRead 00020767 589.PRO
Listmode Replay: New Protocol
Analysis Date: 02-Mar-2021, 17:52:50
Settings File: ƒ „ .PRO, 20-Jan-2021, 16:45:06
Listmode File: 20210120 • ，231-2 002 NoRead 00020767 589.LMD

Run Date: 20-Jan-21, 17:04:59
Sample ID: 20210120 • ，231-2
User ID: user1
Acquisition Time/Events: 52.1s / 15000 (PROTOCOL)

Instrument SN: AU18113 Software Version: Navios 1.1

(30000) [A] FL1 INT LOG/FL3 INT LOG

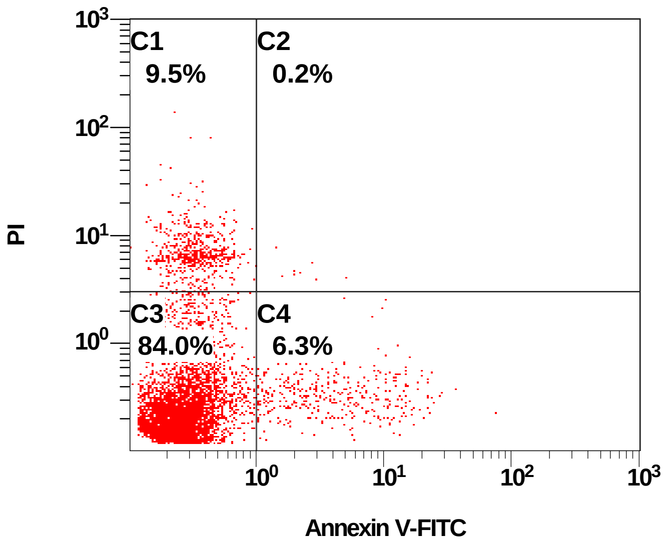

Statistical Analysis

PROGRAM INFORMATION

File:- 20210120 • , 231-2 002 NoRead 00020767 589.LMD					
Gate:- A [A]					
Compensation:-					
Region	Number	%Total	%Gated	X-Mean	Y-Mean
ALL	11811	78.74	100.00	0.655	1.09
ALL	11811	78.74	100.00	0.655	300
ALL	11811	78.74	100.00	1.09	300
C1	1120	7.47	9.48	0.351	8.24
C2	23	0.15	0.19	3.43	5.79
C3	9923	66.15	84.01	0.283	0.331
C4	745	4.97	6.31	5.99	0.374

File:- 20210120 • , 231-2 002 NoRead 00020767 589.LMD					
Gate:- Ungated					
Compensation:-					
Region	Number	%Total	%Gated	X-Mean	Y-Mean
ALL	15000	100.00	100.00	447	483
A	11811	78.74	78.74	300	347
