# Supplementary material for: High HSPB1 expression predicts poor clinical outcomes and correlates with breast cancer metastasis
Source: BMC Cancer. 2023 Jun 3;23:501. doi: 10.1186/s12885-023-10983-3 (PMC10239126; doi:10.1186/s12885-023-10983-3)
Supplement: Supplementary file 6 — Additional file 6. [file 12885_2023_10983_MOESM6_ESM.pdf]

Institution:
Protocol :20210201 • , SK-3 7-ADD NoRead 00020992 814.PRO
Listmode Replay: New Protocol
Analysis Date: 02-Mar-2021, 18:38:02
Settings File: Settings modified during acquisition, N/A
Listmode File: 20210201 • , SK-3 7-ADD NoRead 00020992 814.LMD

Run Date: 01-Feb-21, 16:04:18
Sample ID: 20210201 • , SK-3
User ID: user1
Acquisition Time/Events: 46.4s / 8002 (MANUAL)

Instrument SN: AU18113 Software Version: Navios 1.1

[A] FL2 INT LOG/FL4 INT LOG

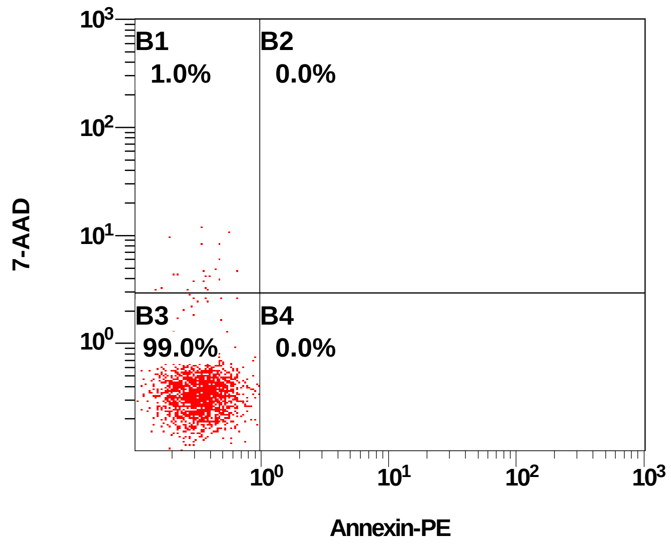

Statistical Analysis

PROGRAM INFORMATION

File:- 20210201 • , SK-3 7-ADD NoRead 00020992 814.LMD

Gate:- A [A]

Compensation:-

Region	Number	%Total	%Gated	X-Mean	Y-Mean
ALL	3472	43.39	100.00	0.352	0.437
ALL	3472	43.39	100.00	0.437	473
ALL	3472	43.39	100.00	168	0.352
ALL	3472	43.39	100.00	168	473
ALL	3472	43.39	100.00	168	0.437
ALL	3472	43.39	100.00	0.352	473
B1	34	0.42	0.98	0.361	6.48
B2	0	0.00	0.00	0	0
B3	3437	42.95	98.99	0.352	0.377
B4	1	0.01	0.03	1.01	0.288
C1	0	0.00	0.00	0	0
C2	0	0.00	0.00	0	0
C3	277	3.46	7.98	2.42	0.335
C4	3195	39.93	92.02	183	0.353
D1	38	0.47	1.09	1.51	5.96
D2	4	0.05	0.12	10.5	3.82
D3	199	2.49	5.73	2.35	0.503
D4	3231	40.38	93.06	181	0.363

File:- 20210201 • , SK-3 7-ADD NoRead 00020992 814.LMD

Gate:- Ungated

Compensation:-

Region	Number	%Total	%Gated	X-Mean	Y-Mean
ALL	8002	100.00	100.00	722	653
A	3472	43.39	43.39	473	431
