# Supplementary material for: High HSPB1 expression predicts poor clinical outcomes and correlates with breast cancer metastasis
Source: BMC Cancer. 2023 Jun 3;23:501. doi: 10.1186/s12885-023-10983-3 (PMC10239126; doi:10.1186/s12885-023-10983-3)
Supplement: Supplementary file 7 — Additional file 7. [file 12885_2023_10983_MOESM7_ESM.pdf]

Institution:

Protocol :20210201 • , SK-4 ƒ „ 3 NoRead 00021000 822001.PRO

Listmode Replay: New Protocol

Analysis Date: 02-Mar-2021, 18:43:13

Settings File: Settings modified during acquisition, N/A

Listmode File: 20210201 • , SK-4 ƒ „ 3 NoRead 00021000 822.LMD

Run Date: 01-Feb-21, 16:17:12

Sample ID: 20210201 • , SK-4

User ID: user1

Acquisition Time/Events: 32.3s / 10000 (PROTOCOL)

Instrument SN: AU18113 Software Version: Navios 1.1

[A] FL2 INT LOG/FL4 INT LOG

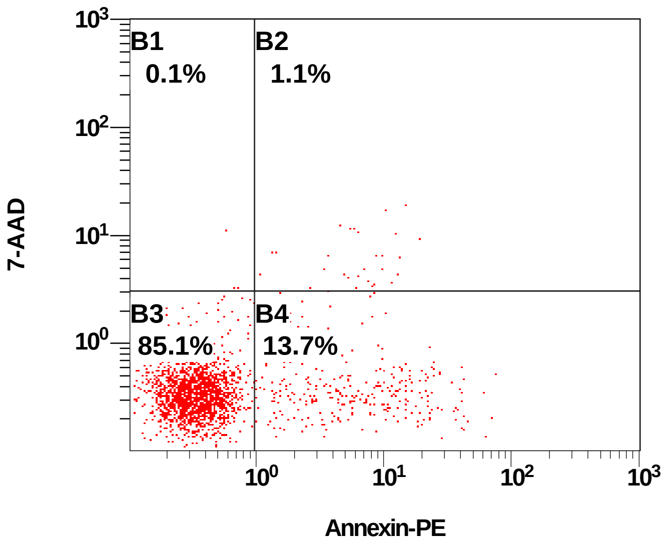

Statistical Analysis

PROGRAM INFORMATION

File:- 20210201 • , SK-4 ƒ „ 3 NoRead 00021000 822.LMD					
Gate:- A [A]					
Compensation:-					
Region	Number	%Total	%Gated	X-Mean	Y-Mean
ALL	4437	44.37	100.00	1.72	0.472
ALL	4437	44.37	100.00	154	1.72
ALL	4437	44.37	100.00	154	450
ALL	4437	44.37	100.00	154	0.472
ALL	4437	44.37	100.00	0.472	450
ALL	4437	44.37	100.00	1.72	450
B1	6	0.06	0.14	0.601	4.62
B2	50	0.50	1.13	7.37	6.64
B3	3775	37.75	85.08	0.357	0.377
B4	606	6.06	13.66	9.79	0.509
C1	178	1.78	4.01	2.46	10.5
C2	247	2.47	5.57	7.56	16.1
C3	257	2.57	5.79	2.25	1.19
C4	3755	37.55	84.63	181	0.405
D1	59	0.59	1.33	1.81	5.82
D2	12	0.12	0.27	5.73	4.88
D3	306	3.06	6.90	2.13	0.676
D4	4060	40.60	91.50	168	0.365

File:- 20210201 • , SK-4 ƒ „ 3 NoRead 00021000 822.LMD					
Gate:- Ungated					
Compensation:-					
Region	Number	%Total	%Gated	X-Mean	Y-Mean
ALL	10000	100.00	100.00	716	622
A	4437	44.37	44.37	450	374
