# Supplementary material for: High HSPB1 expression predicts poor clinical outcomes and correlates with breast cancer metastasis
Source: BMC Cancer. 2023 Jun 3;23:501. doi: 10.1186/s12885-023-10983-3 (PMC10239126; doi:10.1186/s12885-023-10983-3)
Supplement: Supplementary file 8 — Additional file 8. [file 12885_2023_10983_MOESM8_ESM.pdf]

Institution:
Protocol :20210201 • , SK-4 ƒ „ 2 NoRead 00020999 821.PRO
Listmode Replay: New Protocol
Analysis Date: 02-Mar-2021, 18:42:42
Settings File: Settings modified during acquisition, N/A
Listmode File: 20210201 • , SK-4 ƒ „ 2 NoRead 00020999 821.LMD

Run Date: 01-Feb-21, 16:16:18
Sample ID: 20210201 • , SK-4
User ID: user1
Acquisition Time/Events: 25.8s / 10000 (PROTOCOL)

Instrument SN: AU18113 Software Version: Navios 1.1

[A] FL2 INT LOG/FL4 INT LOG

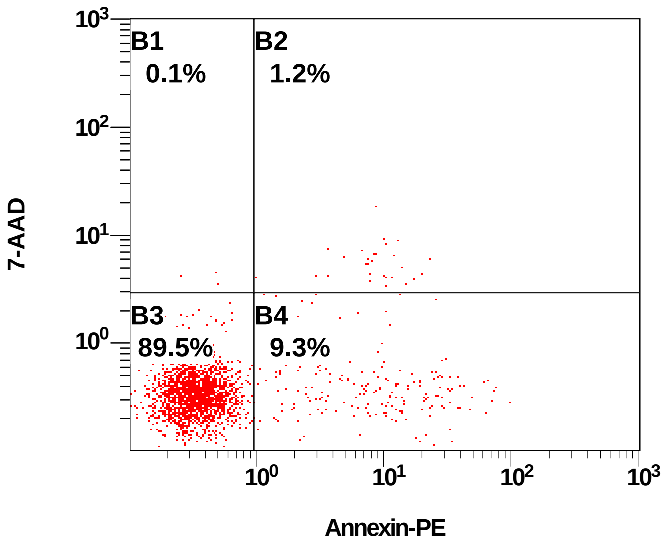

Statistical Analysis

PROGRAM INFORMATION

File:- 20210201 • ，SK-4 ƒ „2 NoRead 00020999 821.LMD

Gate:- A [A]

Compensation:-

Region	Number	%Total	%Gated	X-Mean	Y-Mean
ALL	4267	42.67	100.00	1.66	0.446
ALL	4267	42.67	100.00	166	1.66
ALL	4267	42.67	100.00	166	478
ALL	4267	42.67	100.00	166	0.446
ALL	4267	42.67	100.00	0.446	478
ALL	4267	42.67	100.00	1.66	478
B1	4	0.04	0.09	0.394	4.02
B2	50	0.50	1.17	10	5.88
B3	3817	38.17	89.45	0.353	0.364
B4	396	3.96	9.28	13.2	0.514
C1	84	0.84	1.97	2.82	11
C2	246	2.46	5.77	7.9	18.6
C3	95	0.95	2.23	2.69	1.02
C4	3842	38.42	90.04	184	0.389
D1	35	0.35	0.82	2.3	4.74
D2	29	0.29	0.68	5.87	5.91
D3	92	0.92	2.16	2.41	0.801
D4	4111	41.11	96.34	172	0.363

File:- 20210201 • ，SK-4 ƒ „2 NoRead 00020999 821.LMD

Gate:- Ungated

Compensation:-

Region	Number	%Total	%Gated	X-Mean	Y-Mean
ALL	10000	100.00	100.00	691	600
A	4267	42.67	42.67	478	398
