# Supplementary figures and images for: High HSPB1 expression predicts poor clinical outcomes and correlates with breast cancer metastasis
Source: BMC Cancer. 2023 Jun 3;23:501. doi: 10.1186/s12885-023-10983-3 (PMC10239126; doi:10.1186/s12885-023-10983-3)

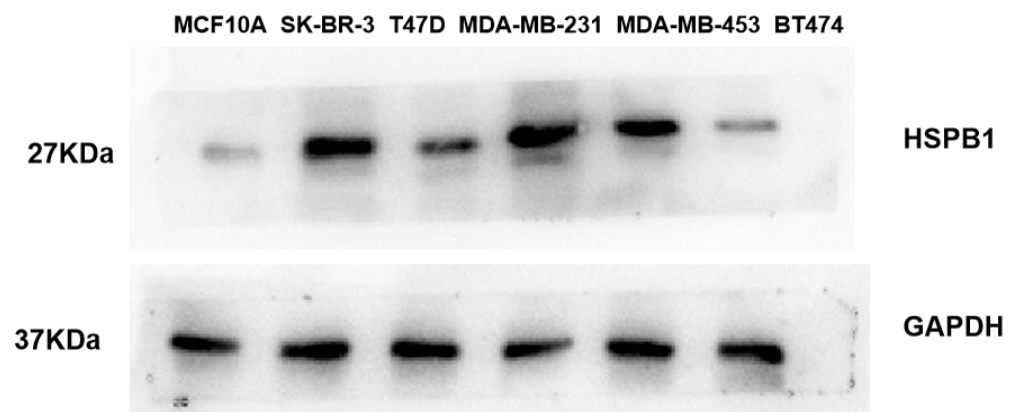

Supplement: Supplementary file 9 — Additional file 9. [file 12885_2023_10983_MOESM9_ESM.pdf]

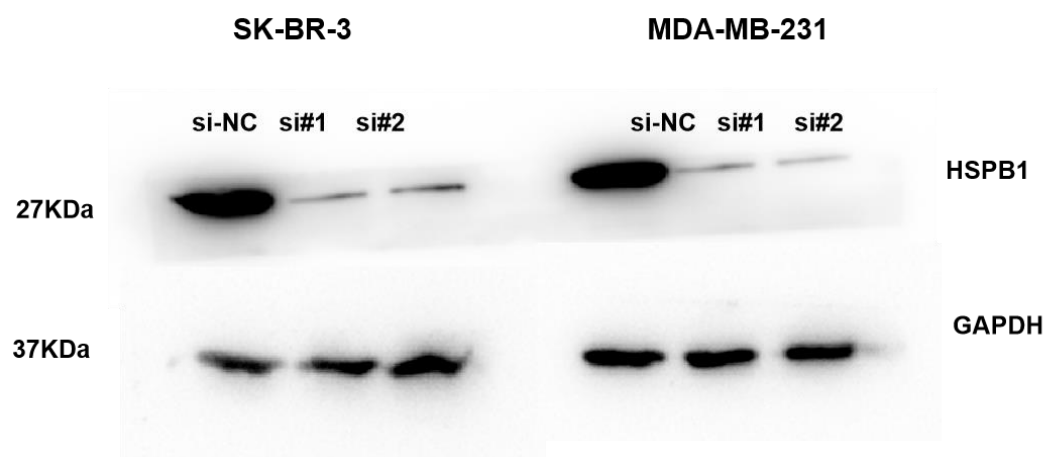

Supplement: Supplementary file 10 — Additional file 10. [file 12885_2023_10983_MOESM10_ESM.pdf]

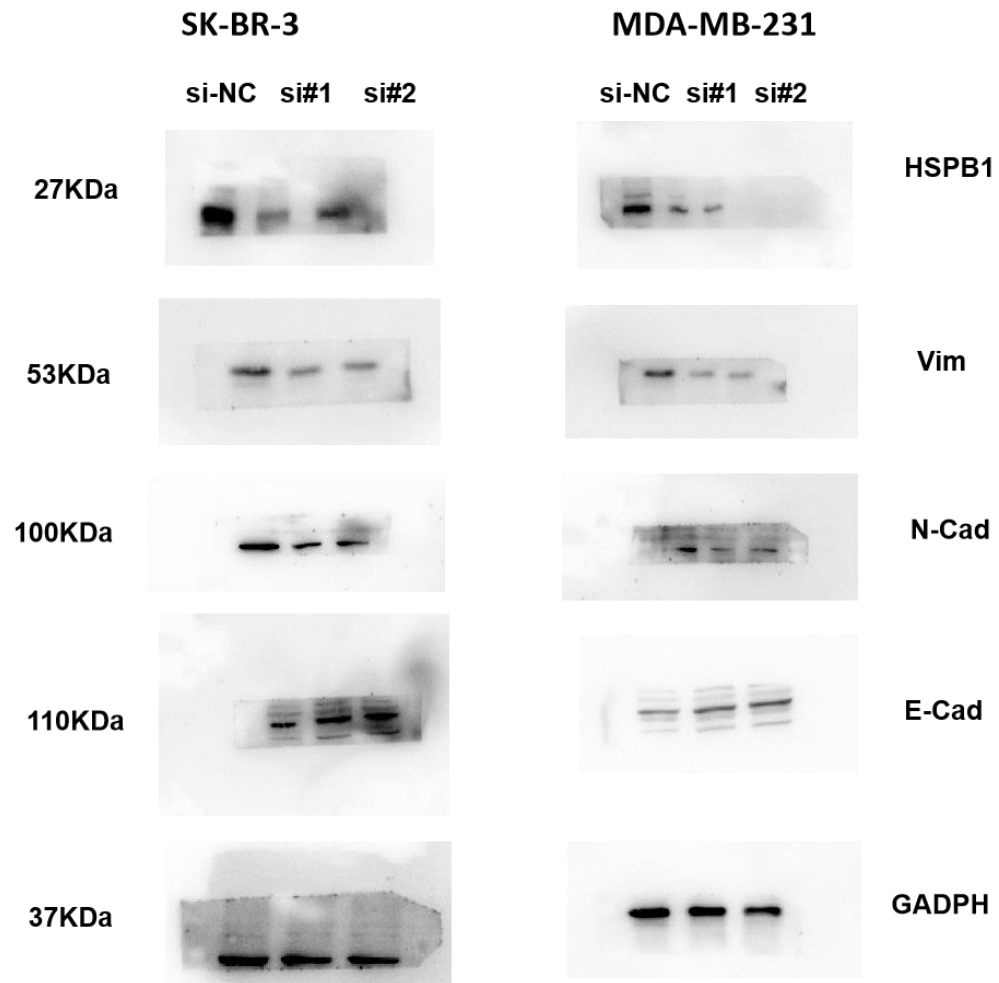

Supplement: Supplementary file 11 — Additional file 11. [file 12885_2023_10983_MOESM11_ESM.pdf]
